# Oral Health Promotion in Infants and Children: Models and Long-Term Effectiveness

**DOI:** 10.1155/2014/385687

**Published:** 2014-04-13

**Authors:** Gajanan Kulkarni

**Affiliations:** Pediatric and Preventive Dentistry, Faculty of Dentistry, University of Toronto, 124 Edward Street, Room 455D, Toronto, ON, Canada M5G 1G6

The concepts of anticipatory guidance, dental home, and the year one dental visit in the context of oral health in children are slowing permeating the dental community. The above principles are now taught in dental schools, especially at the graduate level in specialty pediatric dentistry programs. Various dental associations, societies, and advocacy bodies have adopted those principles and advocate for them to varying degrees. While the value of oral health promotion in infants and young children is widely recognized, there is a diversity of approaches to the same. To date, a comprehensive overview of the various models, best practices, or the benchmarks for assessing their effectiveness has not been undertaken.

In this special issue, dental and nondental clinicians, health promoters, and investigators have contributed to original research articles as well as proposals that document practices, experiences, and evaluation of outcomes related to oral health promotion in infants and young children from around the world. Some of the articles document the long-term effectiveness of their oral health promotion activities or models with clearly identifiable clinical outcomes.

The issue highlights several facts. There is a diversity of approaches employed by a diversity of professionals. The goals, methodology, and outcome measures vary greatly. For example, the ages of the children at which these efforts ought to be directed vary. The scope of the oral health promotion activities ranges from simply providing education to parents and caregivers to provision of preventive services such as the application of fluoride varnishes. Another issue that comes to light is whether health promotion activities should primarily be aimed at caries prevention or if they should provide comprehensive anticipatory guidance which includes education on matters such as prevention of malocclusions in children due to habits, prevention of trauma, and avoidance of other risk behaviors that can predispose children to oral problems. With the advent of newer digital technologies and platforms for dissemination of information, the scope and reach of such activities are bound to evolve. Teledentistry should allow for well-established programs with documented clinical effectiveness to reach parts of the world where childhood oral disease is endemic and access to care is still an issue. There is a need for the development of oral health promotion models deliverable through modern technologies.

There is great paucity of the literature documenting the effectiveness of such programs, both in the short and especially the long term. Moreover, the relationship between oral health in infancy and that in adulthood has yet to be systematically explored. While it is reasonable to conjecture that the establishment of risk factors very early in life might have long-lasting and indeed life-long effects, this has not been explored and documented. Without such evidence it would be difficult to convince governmental or non-governmental agencies to fund such programs and educational institutions to undertake training of health promoters.

This special journal issue is a starting point for developing a consensus around this important pediatric health topic.

*Gajanan Kulkarni* *Gajanan Kulkarni*

